# Regional analysis of cerebral hemodynamic changes during the head-up tilt test in Parkinson’s disease patients with orthostatic intolerance

**DOI:** 10.1117/1.NPh.7.4.045006

**Published:** 2020-10-29

**Authors:** Zephaniah Phillips, Jung Bin Kim, Seung-Ho Paik, Shin-Young Kang, Nam-Joon Jeon, Beop-Min Kim, Byung-Jo Kim

**Affiliations:** a Korea University, Department of Bio-Convergence Engineering, Seoul, Republic of Korea; b Korea University Anam Hospital, Department of Neurology, Seoul, Republic of Korea; c KLIEN Inc., Seoul Biohub, Seoul, Republic of Korea; d Korea University Anam Hospital, Neurophysiology Laboratory, Seoul, Republic of Korea; e Korea University Anam Hospital, Brain Convergence Research Center, Seoul, Republic of Korea; f Korea University, BK21 FOUR Program in Learning Health Systems, Seoul, Republic of Korea

**Keywords:** autonomic dysfunction, automated anatomical labeling, diffuse optical tomography, head-up tilt, orthostatic hypotension, Parkinson’s disease

## Abstract

**Significance:** Cerebral oxygenation changes in the superior, middle, and medial gyri were used to elucidate spatial impairments of autonomic hemodynamic recovery during the head-up tilt table test (HUTT) in Parkinson’s disease (PD) patients with orthostatic intolerance (OI) symptoms.

**Aim:** To analyze dynamic oxygenation changes during the HUTT and classify PD patients with OI symptoms using clinical and oxygenation features.

**Approach:** Thirty-nine PD patients with OI symptoms [10: orthostatic hypotension (PD-OH); 29: normal HUTT results (PD-NOR)] and seven healthy controls (HCs) were recruited. Prefrontal oxyhemoglobin (HbO) changes during the HUTT were reconstructed with diffuse optical tomography and segmented using the automated anatomical labeling system. Decision trees were used for classification.

**Results:** HCs and PD-NOR patients with positive rates of HbO change (PD-POS) showed the greatest HbO recovery in the superior frontal gyrus (SFG) during tilt. PD-OH and PD-NOR patients with negative rates of HbO change (PD-NEG) showed asymmetric reoxygenation. The classification accuracy was 89.4% for PD-POS versus PD-NEG, 71% for PD-NOR versus PD-OH, and 55.8% for PD-POS versus PD-NEG versus PD-OH. The oxygenation features were more discriminative than the clinical features.

**Conclusions:** PD-OH showed decreased right SFG function, which may be associated with impaired compensatory autonomic responses to orthostatic stress.

## 1 Introduction

The head-up tilt test (HUTT) with simultaneous blood pressure (BP) monitoring has been widely used to diagnose orthostatic hypotension (OH) and it comprises three phases: dynamic tilt (supine to 70° tilt), static tilt (remain tilted at 70°), and post-tilt (return to supine).^1^^–^^5^ OH is one etiology of orthostatic intolerance (OI) diagnosed based on a reduction in BP within 3 min of the static tilt phase. However, the HUTT has poor reproducibility and low sensitivity.^6^^,^^7^ Given that OH is common among patients with Parkinson’s disease (PD), early and accurate diagnosis is crucial for providing optimal intensive care to them.^8^^–^^11^ Several studies have explored alternative biomarkers to diagnose OH and overcome the limitations of HUTT.^12^^–^^14^

Optical methods, such as near-infrared spectroscopy (NIRS) and diffuse optical tomography (DOT), have demonstrated accuracy and convenience for real-time monitoring of cerebral oxygenation, particularly during postural changes.^15^^–^^17^ In our recent DOT study, we found that the rate of oxyhemoglobin (HbO) changes from the dynamic to the static tilt phase may be a sensitive marker for differentiating healthy controls (HCs) from PD patients with OH (PD-OH).^18^ Our previous study also suggested that DOT measurements provide information on cerebral reoxygenation that cannot be gleaned from conventional BP monitoring during HUTT, particularly in patients with PD who have normal HUTT results (PD-NOR).^18^

The regional analysis of the brain in PD-OH patients showed that specific cerebral structures are involved in autonomic control. Pathological studies have shown that neurodegenerative processes associated with alpha-synuclein pathology along the central structures are responsible for autonomic control, which may have implications for the pathophysiological mechanism underlying OH in patients with PD.^19^^,^^20^ However, most studies only observed patients during the static phases in a resting-state supine position; therefore, none of them have provided information about changes in dynamic cerebral oxygenation during tilting or standing.^21^^,^^22^ Due to their robustness, optical-based monitoring tools are considered suitable for additionally monitoring cerebral oxygenation, along with BP changes, during dynamic tilting. However, previous studies did not focus on the autonomic dysfunction associated with PD,^23^^–^^25^ or they did not compensate for regional hemodynamic changes across a wide area of the brain during dynamic tilting.^13^^,^^18^ Pathological changes in the central autonomic network (CAN) also play a key role in PD-OH; thus, investigating the central regions associated with OH during dynamic and static tilts in patients with PD may further elucidate the pathophysiological mechanisms underlying autonomic dysfunction in PD patients.

Few studies have investigated the central regions that are associated with PD-OH;^21^^,^^22^ therefore, we extended our previous DOT study to include a regional analysis of cerebral oxygenation across the frontal area during the dynamic and static tilt phases of HUTT in PD patients showing OI symptoms.^18^ To our knowledge, no study has localized the areas of altered cerebral oxygenation during HUTT in patients with PD. We aimed to analyze the dynamic changes in oxygenation during tilting according to the neuroanatomical labels defined by the Automated Anatomical Labeling (AAL) system, which is a parcellation of the brain that has been widely used in neuroimaging research, including DOT studies,^26^^,^^27^ to identify anatomical regions of interest.^28^ Additionally, we attempted to use a machine learning approach to accurately classify PD patients with OI symptoms and analyze the interplay of various features, including oxygenation-based features, clinical metrics related to PD, and demographic information, during the classification process. This work sought to demonstrate that DOT can be used to monitor changes in cerebral oxygenation during dynamic and static tilting based on anatomical guidance and selective cerebral regions contribute to the accuracy of the classification of patients with PD who show OI symptoms.

## 2 Methods

### 2.1 Subjects

PD patients who showed symptoms of OI were recruited for the present study. As such, the subject pool was the same as that used in our prior study.^18^ Based on the conclusions presented in our prior analysis,^18^ the extended analysis of localized cerebral oxygenation was performed on the same subjects to determine whether our previous findings held at a localized level and if other localized hemodynamic trends existed within the same subject pool. PD was diagnosed based on the criteria by the United Kingdom Parkinson’s Disease Society Brain Bank.^29^ Patients with cognitive impairments, those unable to complete autonomic function tests (AFTs), and those unable to complete questionnaires without assistance were excluded from this study, as were those with medical conditions that could affect the AFT results (e.g., cardiac arrhythmia). Motor function in patients with PD was assessed using the Unified Parkinson’s Disease Rating Scale (UPDRS) Part III scores during the “off” period before AFT.^30^ Hoehn and Yahr (H&Y) staging was used to categorize the global severity of PD.^31^ Cognitive function was measured using the Mini-Mental State Exam (MMSE) and Montreal Cognitive Assessment (MoCA) at recruitment. The programming and execution of motor function were evaluated based on the freezing of gait and impaired gait initiation. Non-motor symptoms, such as rapid eye movement sleep behavior disorder, constipation, and anosmia, were also investigated. Written informed consent was obtained from all enrolled patients. The present study also conformed to the principles outlined in the Declaration of Helsinki, and it was reviewed and approved by the institutional review board.

### 2.2 Autonomic Function Tests

Previous studies have detailed the procedure of the AFT.^13^^,^^14^^,^^18^ Briefly, all participants were requested to abstain from medications, alcohol, or coffee, which could affect autonomic function, for at least 24 h before the test. The tests were performed in the following sequence: (1) quantitative sudomotor axon reflex test; (2) heart rate response to deep breathing; (3) Valsalva maneuver; (4) HUTT. The Composite Autonomic Severity Score (CASS), a validated measurement of the severity of autonomic dysfunction, was obtained from the AFTs.^32^ The patients were classified as having OH if there was a reduction in systolic BP of at least 20 mmHg or diastolic BP of at least 10 mmHg within 3 min of the HUTT.^3^ Since OH was the only OI symptom measured within the patient group, all other patients were classified as PD-NOR. A normal HUTT result implied that the patient’s BP changes did not meet the requirements for OI diagnosis. If specific symptoms were observed during HUTT, the patients were returned quickly to the supine position. Therefore, the total static tilt duration varied between subjects.

The patients in the PD-NOR group were further sub-grouped according to the rate of HbO change from the dynamic tilt phase to the static tilt phase: those with a negative rate of HbO change were allocated to the PD-NEG group, whereas those with a positive rate of HbO change were allocated to the PD-POS group. We previously showed that patients in the PD-NEG group demonstrated HUTT cerebral oxygenation patterns that closely resembled PD-OH, and the PD-POS group was well-matched to the HC group.^18^

### 2.3 Diffuse Optical Tomography

A continuous-wave 108-channel DOT system, comprising 15 detectors and 12 sources, was constructed, and it spanned the entire forehead.^13^^,^^14^^,^^18^^,^^33^ The sources alternated between wavelengths of 760 and 830 nm, with a sampling frequency of 5 Hz for the entire forehead. Before the HUTT began, the clinicians ensured that there was adequate contact for all sources and detectors by verifying light intensity data from a custom-made NIRS software. The probe was disinfected with an alcohol swab before it was applied to the patients. In total, the preparation for the HUTT test lasted for ∼10  min. The bottom of the probe was aligned along the Fp1-FpZ-Fp2 line and secured to the subject’s forehead using a band. The subjects were instructed to avoid large head motions that could mask the hemodynamic signal. The probe was removed once the subject returned to the supine position and laid comfortably.

Of the 108 channels, 40 had a source-detector (SD) distance of 15 mm, 20 had an SD distance of 30 mm, 32 had an SD distance of 36 mm, and 16 had an SD distance of 45 mm. The data were collected and processed offline using MATLAB 2013b (The MathWorks Inc., Natick, Massachusetts). The data pre-processing steps were similar to those in our previous publications.^13^^,^^14^^,^^18^^,^^33^ Light intensity changes were converted to optical density and hemodynamic changes using the modified Beer–Lambert law. A 0.2-Hz low-pass filter was applied to remove system noise, and a 5-point moving average filter was used for smoothing. A wavelet-based denoising method was performed to remove large motion artifacts (Daubechies 5).^33^ For DOT calculations, the forward model was built using AtlasViewer. Linear DOT calculations were performed using spatially variant regularization to increase sensitivity in deeper regions of the brain.^34^^,^^35^

**Fig. 1:**
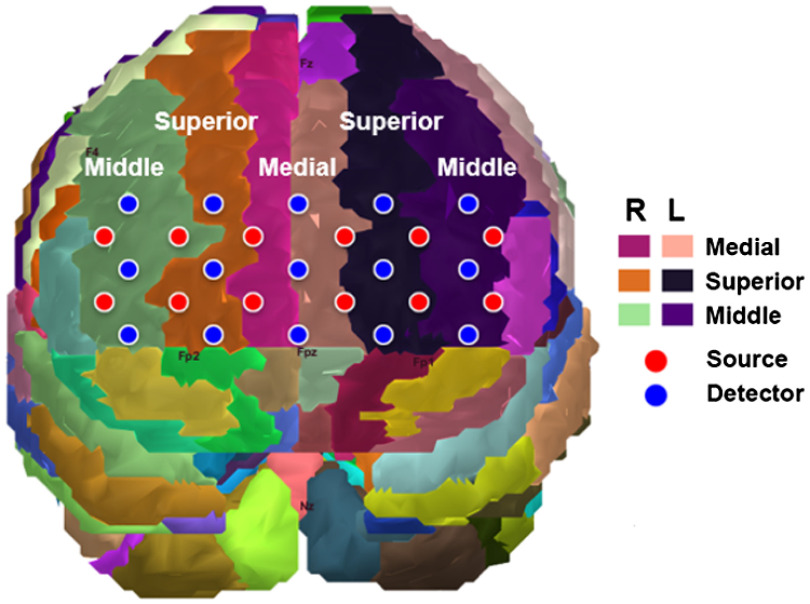
Approximate placement of the sources and detectors of the DOT system within the prefrontal area of the subject, along with the AAL rendered in AtlasViewer.

**Fig. 2:**
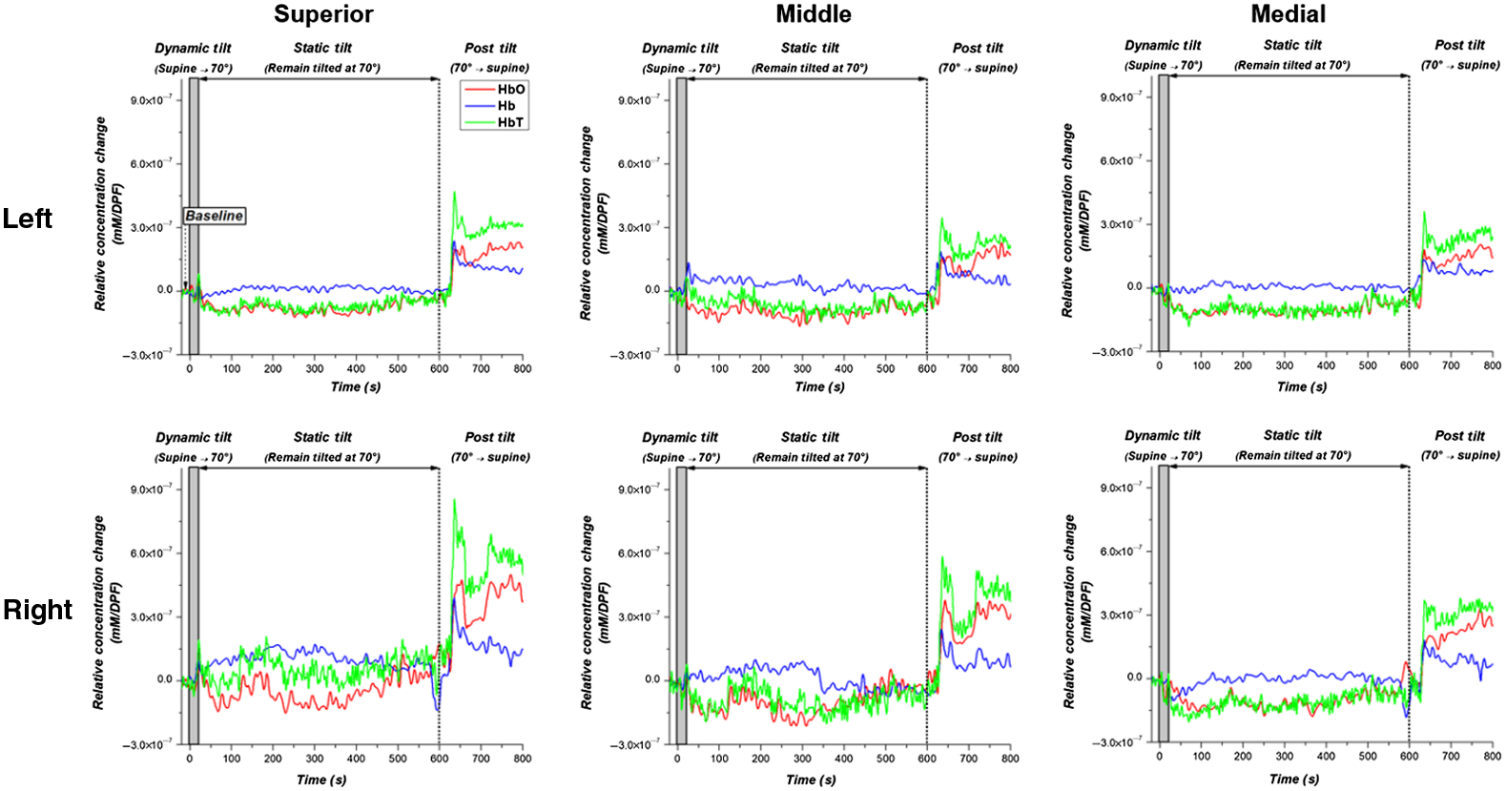
Examples of hemodynamic changes (HbO-red, Hb-blue, and HbT-green) in the left and right superior, middle, and medial gyri during the HUTT in a patient with Parkinson’s disease diagnosed with orthostatic hypotension. The baseline period from which the relative hemodynamics were calculated is indicated on the graph. The three HUTT phases are additionally denoted on the graph: dynamic tilt (supine to 70°), static tilt (remain tilted at 70°), and post-tilt (70° to supine).

Figure 1 shows the DOT probe rendered onto the Colin27 magnetic resonance imaging (MRI) template using AAL labels, which were registered and labeled using AtlasViewer.^36^ Based on this labeling scheme, the hemodynamic changes were spatially averaged over the corresponding gyrus, allowing oxygenation changes of the left and right superior, middle, and medial frontal gyri to be compared. Figure 2 shows the hemodynamic response of each gyrus in one PD-OH patient. The figure indicates (1) the 20-s baseline period when the relative hemodynamic changes were calculated, (2) the dynamic tilt period, (3) the static tilt period, and (4) the post-tilt period. In the patient with PD-OH, there was a continuous reduction in HbO and total hemoglobin (HbT) from the dynamic phase to the static tilt phase (in a majority of the gyri) without reoxygenation to baseline values until the post-tilt period.

### 2.4 Decision Tree Classification

Decision trees are supervised machine learning algorithms that provide a simple and intuitive method for classification. They can also be used to quantify the importance of input features for accurate classification.^37^^,^^38^ In the present work, we implemented the TreeBagger classifier from MATLAB, an ensemble of decision trees with bagging also known as the random forest method.^39^ Since a single decision tree is likely to overfit the dataset, ensemble methods are used to grow multiple decision trees from different subsets of the data. Ensemble methods for NIRS data improve machine learning performance through the combination of several weak learners.^40^ Decision tree classification randomly samples the input data (i.e., bootstrapping) and tests the out-of-bag samples. This randomization process decorrelates individual decision trees to prevent overfitting. In general, it has been approximated that one-third of the instances are left out during decision tree ensemble modeling for testing to compute the out-of-bag accuracy and error.^38^ Additionally, after producing several trees, the ensemble method permutates the values within a feature and calculates the change in error, which in turn, quantifies the importance of a feature (PermutedVarDeltaError function in MATLAB).^24^ In total, 1000 decision trees were generated for three classification trials: (1) PD-NOR versus PD-OH, (2) PD-NEG versus PD-POS, and (3) PD-NEG versus PD-POS versus PD-OH. Since PD-related metrics were not collected for the HCs, we omitted them from the decision tree classification. As input features, we used oxygenation-based features (i.e., DOT-based features) along with clinical metrics related to demographics, PD-severity, and PD-related motor/nonmotor symptoms. These commonly collected clinical metrics have been explained in Sec. 2.1—*Subjects*. For oxygenation-based features, we linearly fitted the average HbO changes of each gyrus from the dynamic tilt phase to the static tilt phase (i.e., 15 to 60 s). Subsequently, we calculated the rate of HbO change. As mentioned, this feature was effective in discriminating between HC, PD-OH, and PD-NOR sub-groups.^18^

## 3 Results

In total, 41 PD patients with OI symptoms and seven HCs (age: 68.1±4.5  years, 3 males) were recruited for the present study. Two patients were excluded from the final analysis because of the low light intensity or large motion artifacts in their DOT data. Based on the HUTT findings, 10 patients were classified as PD-OH (age: 71.9±9.1, 5 male); of 29 patients in the PD-NOR group (age: 68.7±9.2, 20 male), 17 were sub-grouped as PD-POS (age: 70.1±6.2, 13 males) and 12 were sub-grouped as PD-NEG (age: 65.7±12.7, 7 males). The demographics and clinical data of the study participants are given in Table 1. PD-related metrics were not collected from the HCs since they did not show any sign of disease. The PD-OH, PD-NOR, PD-POS, and PD-NEG groups did not statistically differ in age, sex, prevalence of hypertension, H&Y stage, UPDRS part III, MMSE, MoCA, or composite autonomic symptom score. The PD-OH group had a higher CASS than the PD-NOR group (4.6±1.9 versus 3.1±1.8, p=0.049) and the PD-NEG group (4.6±1.9 versus 2.4±1.9, p=0.022).

**Table 1:** Demographics and clinical characteristics.

	HC (n=7)	PD-OH (n=10)	PD-POS (n=17)	PD-NEG (n=12)
Age, years	68.1±4.5	71.9±9.1	70.1±6.2	65.7±12.7
Male, n (%)*	3 (42.9)	5 (50)	13 (76.5)	7 (58.3)
Hypertension, n (%)*	0	4 (40)	10 (58.8)	4 (33.3)
H&Y stage	—	2.3±0.5	2.2±0.9	1.9±0.9
UPDRS part III	—	28.5±11.9	23.1±9.8	24.1±17.2
COMPASS	—	31.7±21.5	11.6±6.6	29±26.3
CASS	—	4.6±1.9	3.6±1.6	2.4±1.9
MMSE	—	25.1±5.1	26.3±3.3	25.5±5.7
MoCA	—	20.6±6.7	22.8±4.6	21.6±8.9
Rate of HbO change ($\times 10^{-4}\;\mathrm{mM}/\mathrm{DPF}$)	7±8.1	−5.6±6.7	8.5±6.3	−9.9±9.9

Abbreviations: CASS, composite autonomic severity score; COMPASS, composite autonomic symptom score; H&Y, Hoehn & Yahr; HUTT, head-up tilt test; MMSE, Mini-Mental State Exam; MoCA, Montreal Cognitive Assessment; OH, orthostatic hypotension; PD, Parkinson’s disease; PD-OH, PD with OH; PD-NOR, PD with normal HUTT; PD-POS, PD with normal HUTT and positive rate of HbO change; PD-NEG, PD with normal HUTT and negative rate of HbO change; UPDRS, Unified Parkinson’s Disease Rating Scale.

Each value represents the mean ± standard deviation. The independent t-test was used to compare the variables between the groups.

Note: * should be used for p value or statistical values hence change * to a

* Chi-square test was performed.

**Fig. 3:**
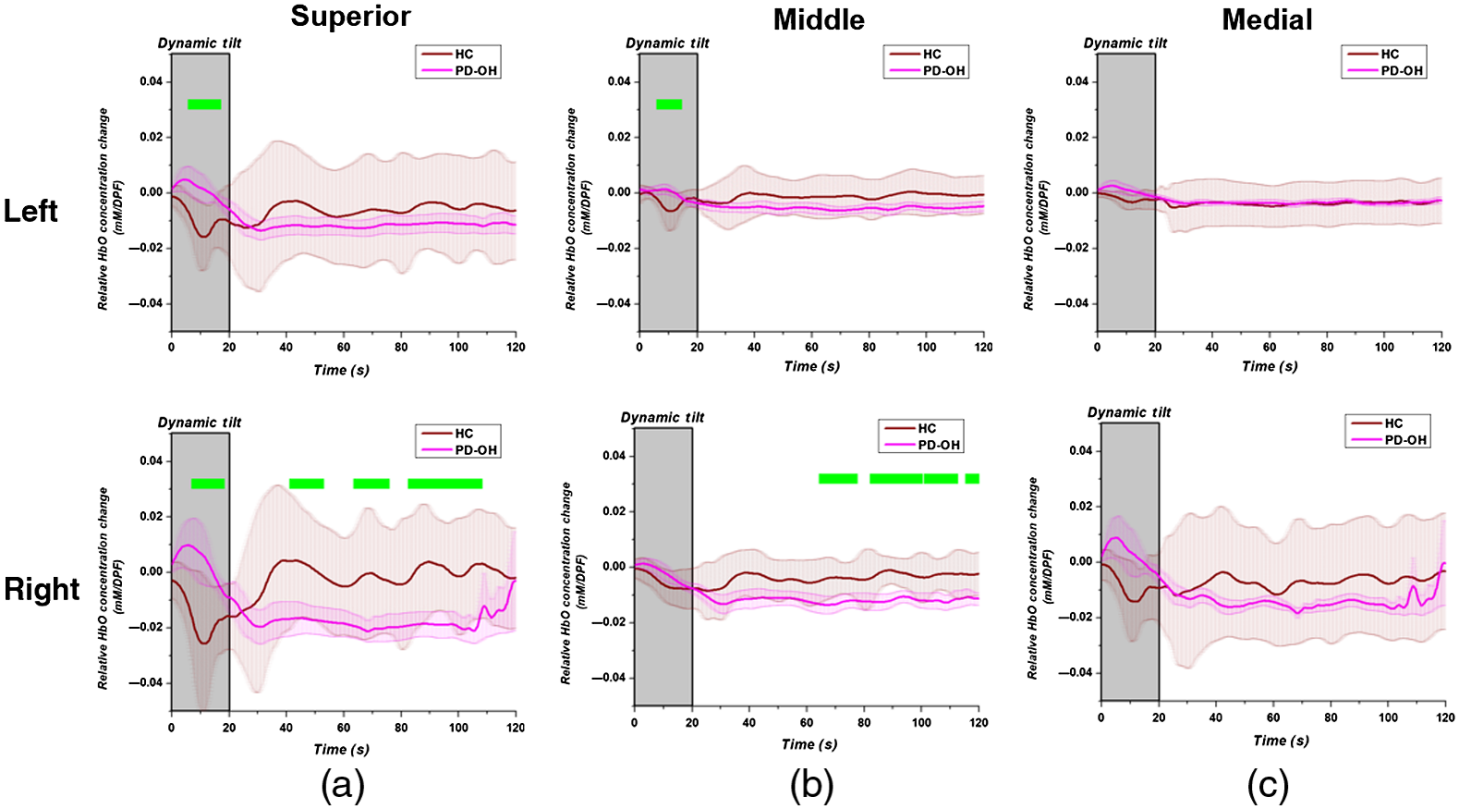
Oxyhemoglobin time-series changes averaged over the (a) superior; (b) middle; and (c) medial frontal gyri for HCs (maroon) and patients with both Parkinson’s disease and orthostatic hypotension (pink). The dynamic tilt phase is highlighted with a gray box. The gyri are separated between the left (upper row) and right (bottom row) hemispheres. Significant differences (p<0.05) between the two groups are denoted with a green bar over the time points.

Our previous study provided a full analysis of the demographics, hemodynamics, and BP response of the subjects.^18^ Because HbO changes were the best discriminators of patients with PD, we performed a regional analysis by averaging the time-series HbO signal of the gyrus. Figure 3 compares the HCs with PD-OH patients based on the average changes in HbO from the dynamic tilt to static tilt phases for the Fig. 3(a) superior, Fig. 3(b) middle, and Fig. 3(c) medial frontal gyri. As shown in Fig. 3, a two-tailed t-test was used to compare the HbO changes at each time point for each subject group. Time points of statistical significance (p<0.05) were highlighted with a bar over the time point. The statistical differences between the HbO changes in the groups largely resulted from the lack of HbO recovery in the PD-OH group during the static tilt, particularly in the right superior and middle frontal gyri.

**Fig. 4:**
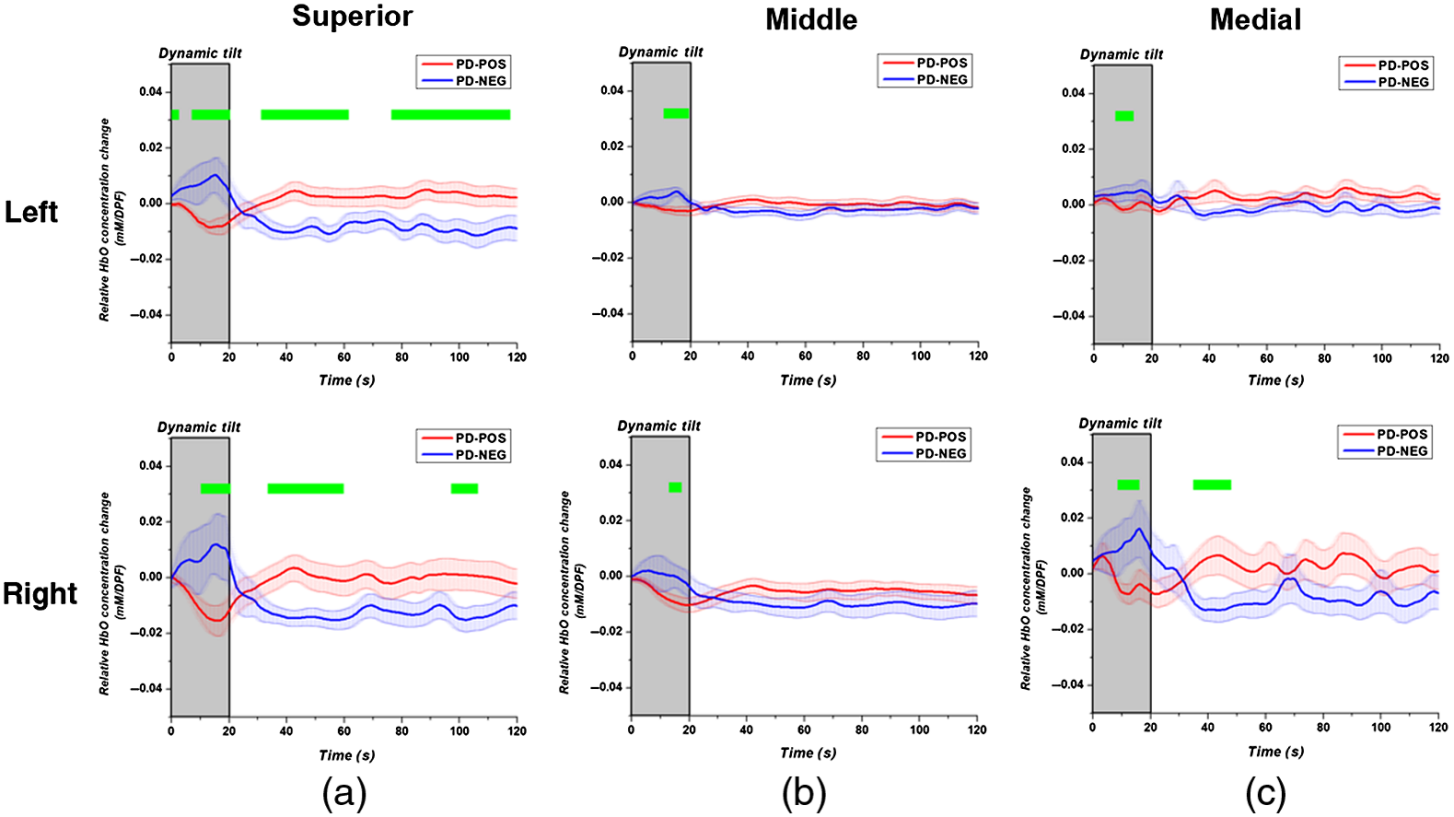
Oxyhemoglobin time-series changes averaged over the (a) superior; (b) middle; and (c) medial frontal gyri in the PD-POS (red) and PD-NEG (blue) sub-groups. The dynamic tilt phase is highlighted with a gray box. The gyri are separated between the left (upper row) and right (bottom row) hemispheres. Significant differences (p<0.05) between the two groups are denoted with a green bar over the time points.

As in Figs. 3 and 4 shows the average time series HbO changes in the PD-POS and PD-NEG sub-groups with a two-tailed t-test performed at each time point. In this case, most of the statistical differences between the HbO changes of the two PD-NOR sub-groups occurred in the left and right superior frontal gyri (SFG) during the dynamic and static tilt phases. When comparing Figs. 3 and 4, both the HC and PD-POS sub-groups showed the most dynamic recovery of HbO in the right SFG, whereas the patients in the PD-OH and the PD-NEG sub-groups showed a continuous reduction in HbO from the dynamic to the static tilt phases.

**Fig. 5:**
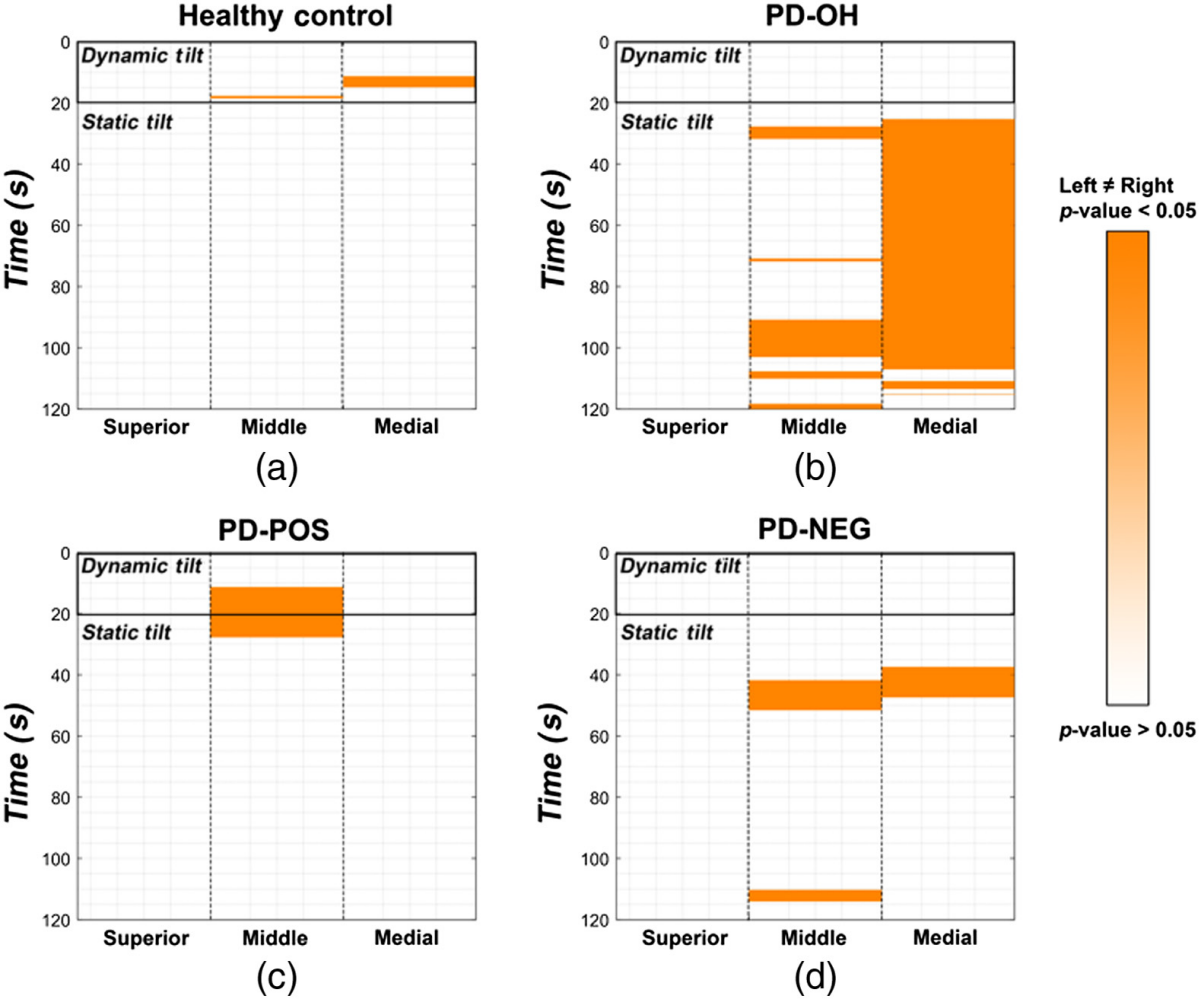
Significant differences (p<0.05; marked in orange) between the left and right superior (first column), middle (second column), and medial frontal gyri (third column) for (a) HCs; (b) PD-OH patients; (c) PD-POS patients; and (d) PD-NEG patients.

Next, we investigated the lateral difference between the gyri in the HCs and patients with PD. Figure 5 shows the time points with a significant difference (p<0.05) between the left and right HbO changes in the frontal gyri. In the HC and PD-POS sub-groups—the two groups that showed recovery of HbO—the HbO changes were not significantly different in either hemisphere for most of the dynamic and static tilt phases. Conversely, in the patients in the PD-OH and the PD-NEG sub-groups—the two groups that did not show a recovery of HbO during static tilt—oxygenation changes in the left and right gyri during the static tilt phase were significantly different. In particular, PD-OH showed asymmetrical reoxygenation in the medial frontal gyrus for most of the static tilt phase and during later periods of the static tilt phase in the middle frontal gyrus.

**Fig. 6:**
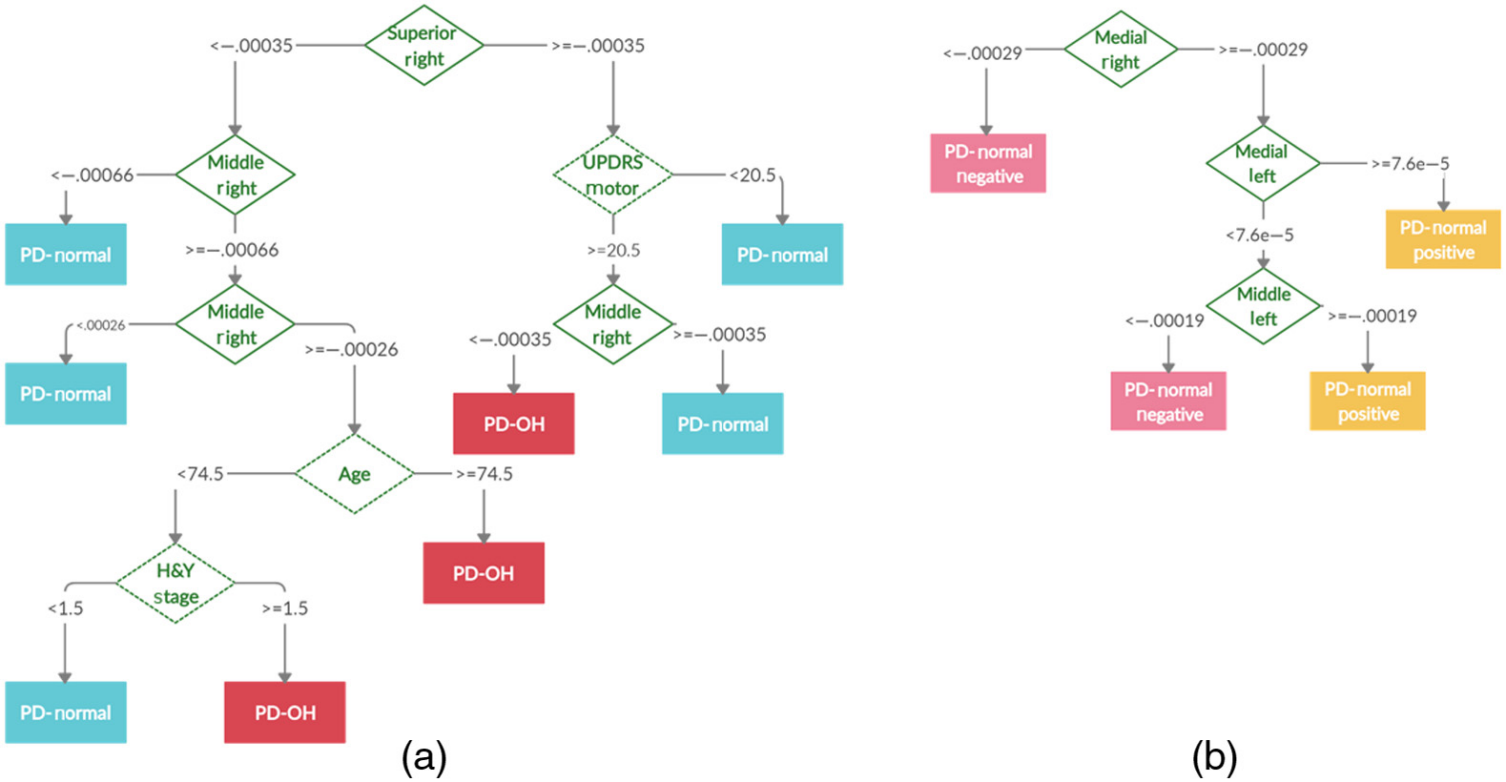
Example of a decision tree used to classify (a) PD-NOR and PD-OH patients and (b) PD-NEG and PD-POS patients. The feature used as a decision node to split the tree is represented with a green diamond. The dashed-line diamond represents a clinical feature and the solid-line diamond represents an oxygenation feature. Each path ends in a leaf node representing a class of patients with Parkinson’s disease.

**Fig. 7:**
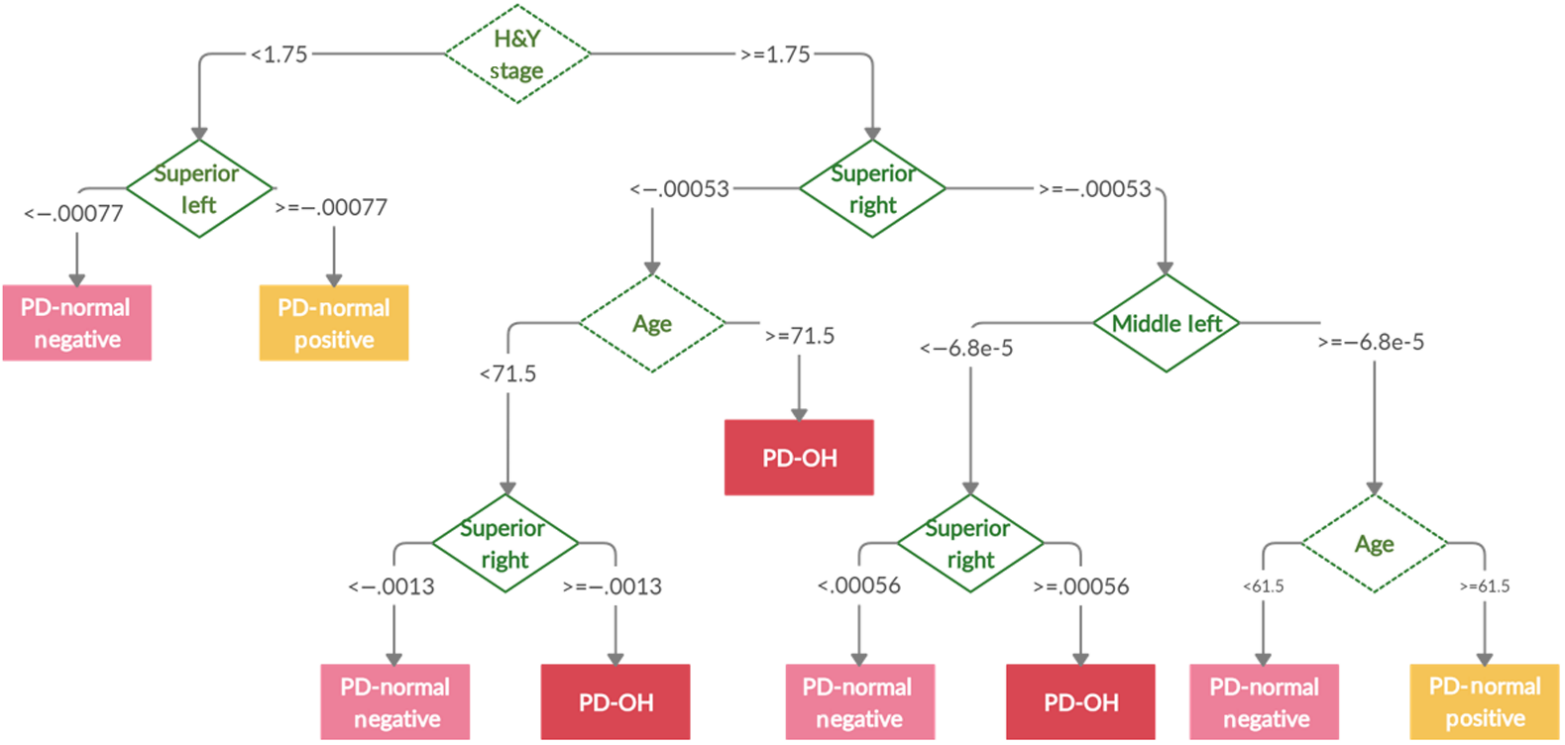
Example of a decision tree used to classify PD-NEG, PD-POS, and PD-OH patients. The feature used as a decision node to split the tree is represented by a green diamond. The dashed-line diamond represents a clinical feature and the solid-line diamond represents an oxygenation feature. Each path ends in a leaf node representing a class of patients with Parkinson’s disease.

Decision tree classification is useful for visualizing the interplay of clinical and oxygenation features to classify PD patients. Figure 6 shows an example of a decision tree created to classify the PD-NOR and PD-OH patients [Fig. 6(a)], and the PD-NEG and PD-POS patients [Fig. 6(b)]. The tree begins at the root node and splits at the decision nodes based on a cutoff value. Each pathway ends at a terminal node that classifies the subject. As shown in Fig. 6(a), the classification of the PD-NOR and PD-OH patients is determined by a combination of oxygenation features related to DOT measurements of the right gyrus-related HbO change, in addition to features related to PD severity (i.e., H&Y stage), motor capability (i.e., UPDRS motor score), and patient demographics (i.e., age). Figure 6(b) shows that the classification of the PD-NEG and PD-POS sub-groups is based on oxygenation features solely related to DOT measurements from lateral gyri. This is expected since the PD-NOR sub-groups were defined according to the DOT results. However, for multi-class classification, it is apparent that oxygenation features and demographic features can be used together by a decision tree to accurately classify PD-OH and PD-NOR patients. Figure 7 shows an example decision tree created for the classification of PD-NEG, PD-POS, and PD-OH patients. The example tree demonstrates that these three classes can be accurately distinguished using oxygenation features primarily of the right gyri, and PD severity (i.e., H&Y stage) and patient demographics (i.e., age).

**Fig. 8:**
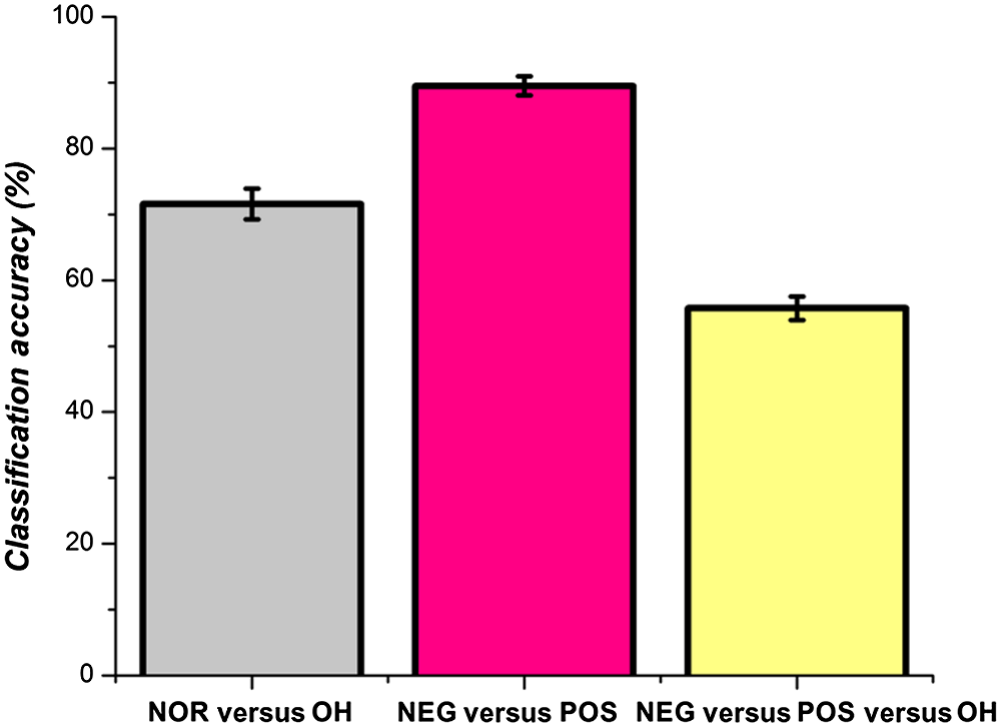
Accuracy of decision tree classification for three different trials: PD-NOR versus PD-OH; PD-NEG versus PD-POS; PD-OH versus PD-NEG versus PD-POS.

Figure 8 shows the average accuracy of the ensemble decision tree method for classifying PD patients. All three trials of classification—PD-NOR versus PD-OH, PD-NEG versus PD-POS, and PD-NEG versus PD-POS versus PD-OH—were above chance level (i.e., two classes = 50% and three classes = 33.3%). The highest accuracy was obtained for the classification of PD-NEG and PD-POS patients (89.4%±1.5%). The PD-NOR and the PD-OH classification showed a reasonably high accuracy above chance level at 71%±2.3%. For the multiclass classification, the accuracy reduced to 55.8%±1.7%. The accuracies for the two-class and three-class classifications were comparable to those of similar ensemble-based machine learning methods based on the NIRS data.^40^ Our results demonstrated that decision tree classification using oxygenation and clinical features could be used for the accurate classification of patients with PD who show OI symptoms.

**Fig. 9:**
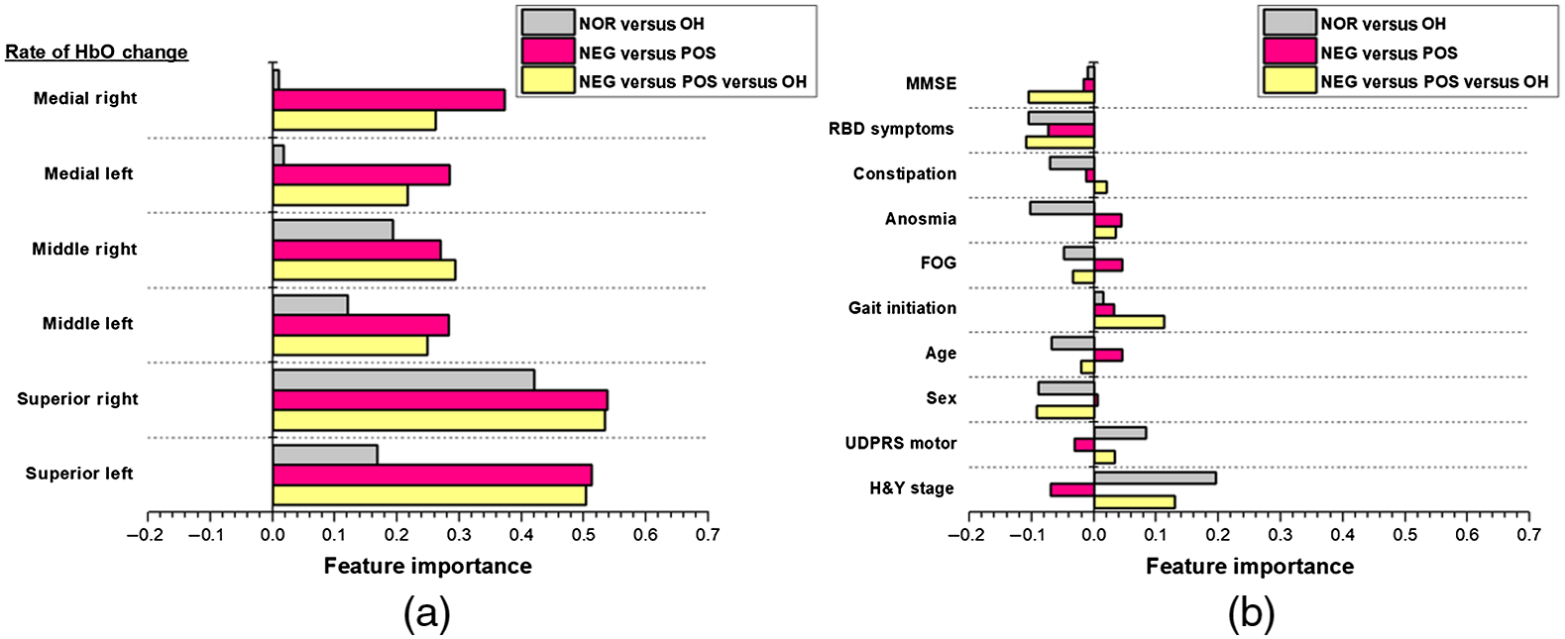
Feature importance according to (a) oxygenation features and (b) clinical features of the three classification trials: PD-NOR versus PD-OH (gray bar); PD-NEG versus PD-POS (pink bar); and PD-NEG versus PD-POS versus PD-OH (yellow bar).

The feature importance metric was calculated for every generated tree using the permutation method and averaged and normalized over the ensemble.^38^^,^^39^ Figure 9 shows the average importance of each feature (oxygenation and clinical) for each of the three classification trials. Oxygenation features had higher feature importance than clinical features in all the three trials. The negative values of the clinical features imply that these features do not enhance the classification accuracy of patients with PD. Of the clinical features, those related to PD severity, H&Y stage, and UDPRS motor showed the highest feature importance in the classification trials involving patients with PD-OH. For the oxygenation features, the SFG (both right and left) showed the highest feature importance in trials involving the PD-NEG and PD-POS sub-groups. Moreover, our decision tree analysis provided evidence that oxygenation features facilitate accurate classification for patients grouped based on DOT data (i.e., PD-POS versus PD-NEG) and those grouped based on BP data (i.e., PD-NOR versus PD-OH). These results demonstrate the advantage of monitoring localized hemodynamic changes using DOT since gyrus-specific oxygenation changes were more important for the classification of patients with PD.

**Fig. 10:**
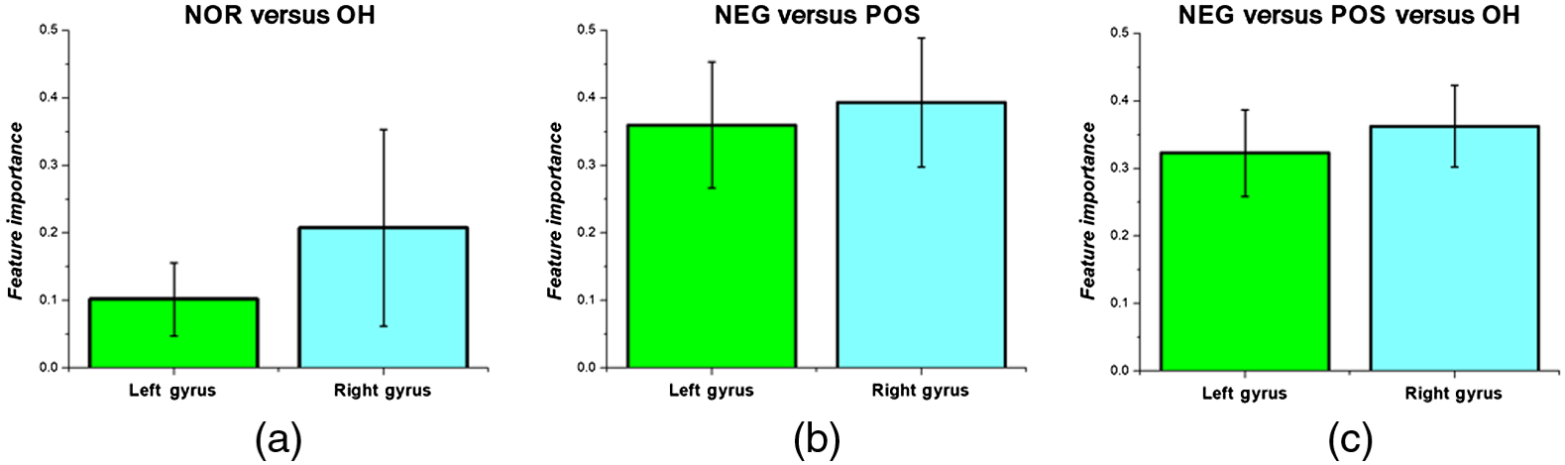
Average feature importance of oxygenation features of the left and right gyri for (a) PD-NOR versus PD-OH; (b) PD-NEG versus PD-POS; and (c) PD-NEG versus PD-POS versus PD-OH.

In Fig. 10, we averaged the feature importance according to the left and right gyri in each of the classification trials to examine whether oxygenation changes from one hemisphere were important for classification. Although the difference was not significant, the right gyri tended to show higher feature importance than the left gyri. The largest difference between the right and left gyri was observed during the classification of the PD-NOR and PD-OH patients.

**Fig. 11:**
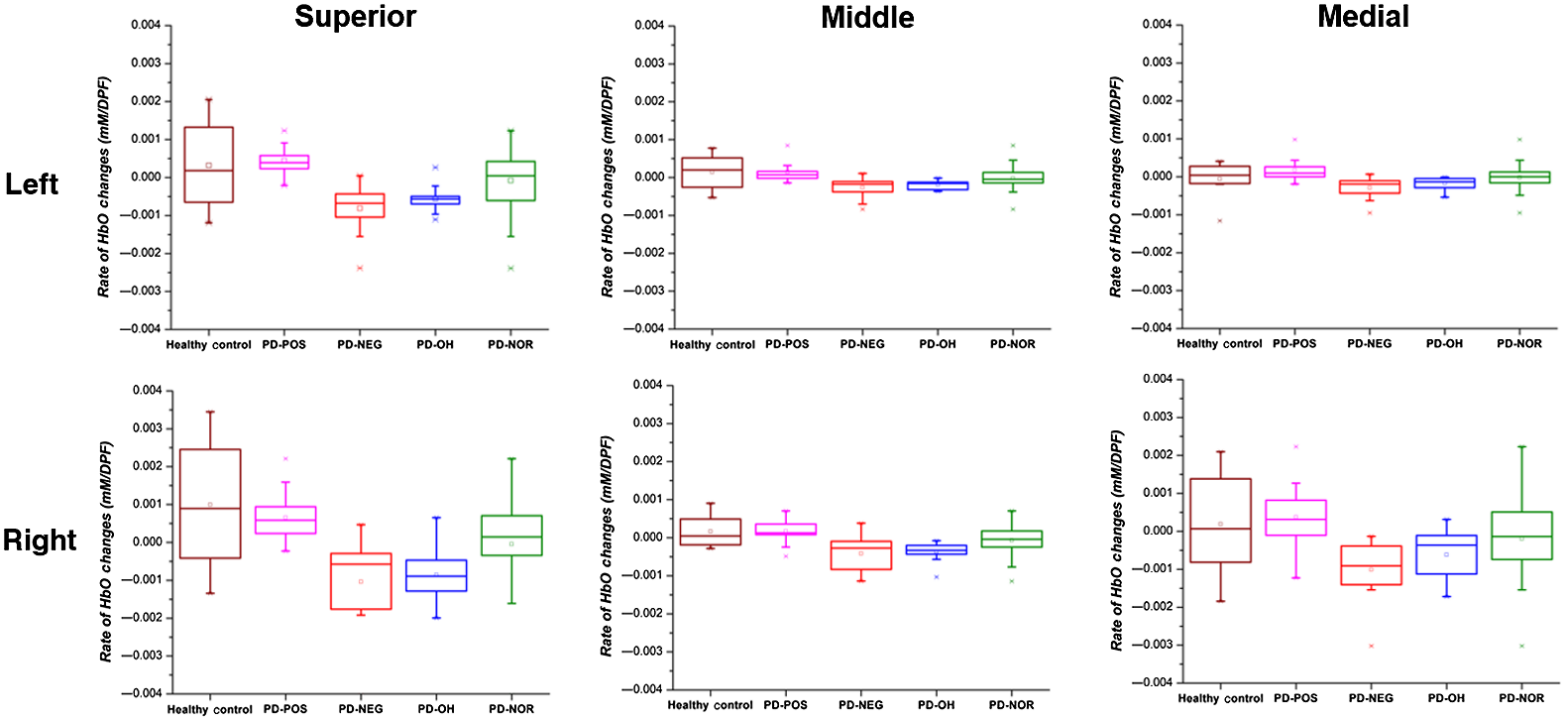
Box plot of the rate of HbO change for HCs, PD-POS, PD-NEG- PD-OH, and PD-NOR groups.

To reinforce our findings of the localized difference between the rates of HbO change, we analyzed the distribution of the rates of change for each of the subject groupings: HCs, PD-POS, PD-NEG, PD-OH, and PD-NOR. Figure 11 shows the box-plot distribution for the rates of HbO change of the groups. The figure shows that the right SFG shows the largest contrast between the groups, particularly between the HCs/PD-POS and PD-OH/PD-NEG. In the right SFG, the distribution of the rates of HbO change for HCs/PD-POS was largely within a positive value range, whereas that of the PD-OH/PD-NEG was largely within the negative value range. In addition, we demonstrate that the PD-POS and PD-NEG sub-groupings facilitate a better discrimination of the groups compared with PD-NOR as a single entity. The distribution of the rates of HbO changes indicates that the SFG, especially the right SFG, is satisfactorily discriminative of the subject groups.

## 4 Discussion

Through the regional DOT analysis of cerebral oxygenation during HUTT, we demonstrated that HbO changes can vary depending on the region and certain gyri may demonstrate a larger effect on classification accuracy in patients with PD who show OI symptoms. The segmentation of the frontal region according to the AAL neuroanatomical labeling scheme showed that the SFG, especially the right SFG, had a more dynamic recovery in the HCs and the PD-POS patients. Conversely, the patients with PD-OH and those with PD-NEG showed a continual reduction in HbO from the dynamic to the static tilt phases in all frontal gyri. Additionally, both the PD-OH and PD-NEG groups showed an increase in HbO during the dynamic tilt process, whereas HCs and PD-POS patients showed a reduction in HbO (Figs. 3 and 4). The increase in HbO during the dynamic tilt for PD-OH and PD-NEG may be attributed to the impaired autonomic nervous system that did not activate compensatory sympathetic activity to maintain cerebral blood flow when standing up, resulting in a gradual decrease in HbO. On the other hand, in HCs and PD-POS, the normal compensatory mechanism stimulates the sympathetic nervous system to maintain cerebral blood, resulting in the recovery of HbO.^41^

The lack of cerebral reoxygenation during the static tilt phase in patients with PD-OH and PD-NEG coincided with the asymmetrical reoxygenation in the middle and medial frontal gyri. Cerebral recovery in the HCs and the PD-POS patients coincided with similar oxygenation changes in the left and right frontal gyri. Additionally, using the decision tree classification, we showed that oxygenation-based features (i.e., rate of HbO change) were more important for the classification of patients with PD with OI symptoms than clinical features. The ensemble method of decision tree classification was a relatively accurate method for distinguishing PD-OH from PD-NEG, PD-POS, and PD-NOR.

Although the precise role of the SFG in hemodynamic response is yet to be clarified, the relationship between SFG function and OI symptoms can be understood in terms of cause and consequence. Perhaps, the impaired autonomic regulatory function in the SFG is the underlies OH in patients with PD. In coordination with the sensory system, the SFG may be involved in working memory, reappraisal function, and self-awareness.^42^^–^^44^ In particular, the anteromedial subregions of the SFG are anatomically and functionally connected with the anterior and mid-cingulate cortices, which are critical components of the cognitive control network, the default mode network (DMN), and the CAN.^45^^,^^46^ The DMN plays a pivotal role in sustaining self-consciousness; it is engaged during self-referential thinking during rest, and the midline core of the DMN is active when making self-relevant and affective decisions.^47^ Moreover, the anterior cingulate cortex is a key component of the CAN, and a central node of the salience network that contributes to a variety of complex functions, such as communication, social behavior, and self-awareness through the integration of sensory, emotional, and cognitive information.^48^^,^^49^ The CAN controls the activity of sympathovagal balance, and it is involved in the modulation of visceral functions, the maintenance of homeostasis, and the adaptation to internal or external challenges.^48^ Given the structural and functional connectivity of the SFG with the DMN and CAN, the SFG is involved in homeostatic controls in response to external changes.^50^^,^^51^ Accordingly, our results showed significant decreased reoxygenation from the dynamic tilt to the static tilt phases in the SFG of patients with PD-OH and PD-NEG compared with those of HCs and patients with PD-POS (Figs. 3 and 4). This could be interpreted as the impairment of the physiological role of the SFG in homeostatic autonomic regulation in response to orthostatic changes.

Alternatively, the reduced oxygenation in the SFG may be a consequence of repeated exposure to hypoperfusion as a consequence of OH. In that case, there would likely be no difference between the left and right patterns of hemodynamic changes. However, we found evidence of unilateral oxygenation changes during HUTT in patients with PD, particularly in those with PD-OH and PD-NEG (Fig. 5). Given that the change in HbO showed laterality, we hypothesize that the difference between the HbO changes in the SFGs of the groups that showed cerebral reoxygenation and those that did not is attributable to the SFG’s functional decrease as a pathophysiological mechanism underlying OH in patients with PD, rather than as a consequence of recurrent exposure to OH. There is evidence of hemispheric lateralization of cardiovascular autonomic control:^48^^,^^52^ intracarotid amobarbital injection in temporal lobe epilepsy shows sympathetic activation with left hemisphere injection and parasympathetic activation with right hemisphere injection.^53^ An intraoperative study before temporal lobectomy in epilepsy patients demonstrated that both BP and heart rate decreased with left insular stimulation, whereas they increased with right insular stimulation.^54^ Moreover, we observed that patients with neurocardiogenic syncope showed right insular atrophy, and the smaller right insular volumes were related to greater reductions in BP during HUTT.^52^ The decrement in HbO without recovery in the right SFG in PD-OH and PD-NEG patients may be interpreted as an impaired sympathetic outflow to compensate for the reduction in oxygen supply during tilting.

Another novel observation was that the rate of HbO change in the right SFG was the most important feature for decision tree classification of patients with PD-OH and those in the PD-NOR sub-group. The rate of HbO change derived from hemodynamic changes averaged over the gyri, combined with demographics and PD-related clinical variables, is important when developing a predictive model. Moreover, these findings suggest that the rate of HbO changes in the right SFG may be important for differential diagnosis, and it is clinically capable of sensitively and quantitatively discriminating autonomic dysfunction, even in PD patients with normal HUTT results.

Several limitations of this study should be noted. First, our sample size was relatively small. Second, the current study was cross-sectional; therefore, our results cannot be used to determine a causal relationship. Further prospective studies incorporating a longitudinal design may provide insights into the causal relationship between the rate of HbO change in specific regions and OH symptoms. Finally, our study did not include PD patients without OH symptoms as a basis of comparison to understand the effect of PD on cerebral hemodynamic changes during HUTT. Therefore, our results cannot be interpreted as PD-specific findings.

Several methodological considerations could be addressed. The hemodynamic modeling and registration of the probe were based on the Colin27 template with AAL labeling since individual patient scans were not available. The use of such brain templates has been recommended and commonly applied in the anatomically guided analysis of DOT data as a feasible alternative to individual MRI scans.^55^^,^^56^ That said, the clinicians tried to align the DOT probe with well-known coordinate points according to the commonly used 10-20 system. We believe that by aligning the bottom of the probe with the Fp1-FpZ-Fp2 line, we obtained a reliable approximation of channel location relative to anatomical features. Finally, more robust machine learning algorithms should be implemented to more accurately classify the patients. In this study, feature extraction from the DOT data was manually performed. By using deep learning techniques, such as convolution neural networks, feature selection and extraction processes can be automated.^57^ Therefore, additional DOT features, in addition to the rate of HbO change, may better distinguish between the patient groups. For our current purpose of analyzing the interplay of features in the decision-making process, the decision tree classification was suitable, as it provided an intuitive and computationally efficient classification method to achieve relatively high classification accuracy.

## 5 Conclusion

In the present work, we used decision trees to classify three types of PD patients based on the HUTT results: those who exhibited OH symptoms based on BP changes, those who had normal HUTT results and showed a positive rate of HbO change, and those who had normal HUTT results but showed a negative rate of HbO change. An ensemble of decision trees was used to accurately classify the PD-patient groups using input features based on DOT measurements and PD- and demographic-related clinical metrics. Oxygenation features derived from DOT measurements and metrics of PD severity had high importance for the accurate classification of patients with PD. We also observed that spatial selectivity was involved in the impairment of autonomic hemodynamic recovery in the right SFG of PD patients with OH, indicating that the right SFG contributes to the pathophysiological mechanism underlying OH in patients with PD. Taken together with the findings of previous studies, the results of this study suggest that the decreased function of the right SFG of patients with PD is associated with the impairment of the compensatory autonomic response to orthostatic stress, which involves sympathetic activation.

## Code, Data, and Materials Availability

Available upon request.
